# Individualized Treatment for Advanced Non-Small Cell Lung Cancer: A Case Report and Literature Review

**DOI:** 10.3389/fonc.2022.916681

**Published:** 2022-05-26

**Authors:** Qianqian Sun, Weiqing Li, Taorui Liu, Huiqin Guo

**Affiliations:** Department of Thoracic Surgery, Beijing Shijitan Hospital, Capital Medical University, Beijing, China

**Keywords:** individualized treatment, NSCLC, surgery, chemotherapy, immunotherapy, targeted therapy

## Abstract

The incidence of lung cancer is high and about 75% of the patients with lung cancer are found in the middle and advanced stage, which has a limited treatment strategy. Non-small cell lung cancer (NSCLC) accounts for about 85% of all lung cancers. In this article, we delineate the treatment process of a middle-aged male patient with advanced-stage lung cancer to explain the significance of individualized chemotherapy combined with immunotherapy and surgery. This patient has extensive bone metastasis with PS scores of 2. After nine cycles of preoperative neoadjuvant chemotherapy, surgery, and two cycles of postoperative adjuvant chemotherapy, the patient achieved complete response (CR) and his PS score was 0. Although there is a standard chemotherapy regimen for lung adenocarcinoma, the treatment effect varies because of individual differences. Comprehensive analysis of the characteristics of patients through a variety of means to develop a precise individualized chemotherapy plan will be a major direction of lung cancer treatment in the future. Additionally, surgical treatment for advanced lung cancer patients after chemotherapy can effectively reduce the primary lesion and prolong the survival time of patients.

## Introduction

Lung cancer is a major health problem worldwide. About 2.1 million people are diagnosed with lung cancer and 1.8 million people die of lung cancer every year (1). Lung cancer is the leading cause of cancer death in the world. So far, there is still no effective method to screen for lung cancer, but studies have shown that annual low-dose chest CT (LDCT) can reduce lung cancer mortality by 20% and overall mortality by 6.7% compared with chest X-ray (CXR) (2). The US Preventive Services Task Force suggests that adults, aged 50 to 80, who have been smoking for 20 years, are still smoking, or have given up smoking for more than 15 years should be screened for lung cancer by LDCT (3). Lung cancer includes non-small cell lung cancer (NSCLC) and small cell lung cancer (SCLC). Adenocarcinoma is the most common pathological type in NSCLC (4), and squamous cell carcinoma ranks second (5). In recent years, the incidence of squamous-cell carcinoma has decreased significantly, which might be related to the lower smoking rate in high-income countries and changes in the composition of cigarettes (6).

Once lung cancer is suspected, diagnosis and staging must be made, because the treatment of lung cancer depends on its subtype and stage. The 5-year survival rates of NSCLC patients with stage I, stage II to stage III, and stage IV were 80%, 13-60%, and 0-10% respectively (7). Surgical resection is the standard treatment in stages I, II, and some IIIA (8). Adjuvant chemotherapy can improve the survival rate of stage II, IIA, or IB patients by 5% - 10%, but it also has side effects (9). For early NSCLC patients who are not suitable for surgery, stereotactic ablation radiotherapy (SABR) can be considered (10). Platinum-based chemotherapy (such as cisplatin and carboplatin) 2-drug regimen is standard for patients with stage IV NSCLC. Although chemotherapy is still indispensable in the treatment of lung cancer, targeted therapy for specific gene mutations has made progress in the past few years (11). The first-generation targeted drugs (gefitinib and erlotinib) and second-generation targeted drugs (afatinib and dacomitinib) for EGFR mutations can significantly improve progression-free survival time (PFS) and overall survival (OS) compared with double platinum chemotherapy (12–17). Crizotinib, a drug targeting ALK mutations, shows better survival than chemotherapy as the first-and second-line therapy in a phase III trial (18–20). In addition, targeted therapy against ROS1, BRAF, NTRK, MET, RET, KRAS, HER2, and other genes has achieved good results in clinical trials.

Compared with the second-line chemotherapy for NSCLC, patients with anti-PD-1 and anti-PD-L1 antibodies always maintain a higher survival rate, which has become an important treatment for primary NSCLC (21–24). In NSCLC, two recognized active immune checkpoints are the CTLA-4 and PD-1 axes. CTLA-4 is usually expressed on CD4 and CD8 positive T lymphocytes and inhibits T cell activation. PD-1 is expressed in T cells, B cells, and NK cells and regulates central and peripheral immune tolerance. The expression of PD-L1 in tumor cells leads to immune escape (25). In the study of non-squamous NSCLC, such as the phase 3 KEYNOTE-189 trial (26–28), PD-1 or PD-L1 antibody combined with platinum chemotherapy is better than chemotherapy alone. Now, patients whose PD-L1 expression is 50% or more can use pembrolizumab or atezolizumab monotherapy, chemotherapy plus immunotherapy, or dual-drug immunotherapy with or without chemotherapy (8). For patients with PD-L1 expression of less than 50%, chemotherapy combined with PD-1 or PD-L1 inhibitors is the standard treatment (8). However, rare toxic effects associated with immunotherapy can happen at any point. It has been reported that immune checkpoint inhibitors (ICIs) can be used for two years (22). If properly treated, immune-related side effects are usually transient, but in some cases, they can be fatal. However, there is a problem with drug resistance in both targeted therapy and immunotherapy. Dealing with the problem of drug resistance is critical for lung cancer treatment.

In recent years, the diagnosis and treatment of lung cancer have made some progress, but the effect is still not satisfactory. Individualized treatment of lung cancer has gradually become a newer trend. In this article, we present a case of a middle-aged male patient with advanced lung adenocarcinoma who underwent a descending surgery after 9 cycles of individualized chemotherapy combined with targeted immunotherapy and continued adjuvant chemotherapy until the condition reached complete response (CR). Here we will elaborate on the choice of treatment and the significance of surgery for patients with advanced lung cancer, to emphasize the importance of individualized therapy.

## Case Presentation

**Figure 1:**
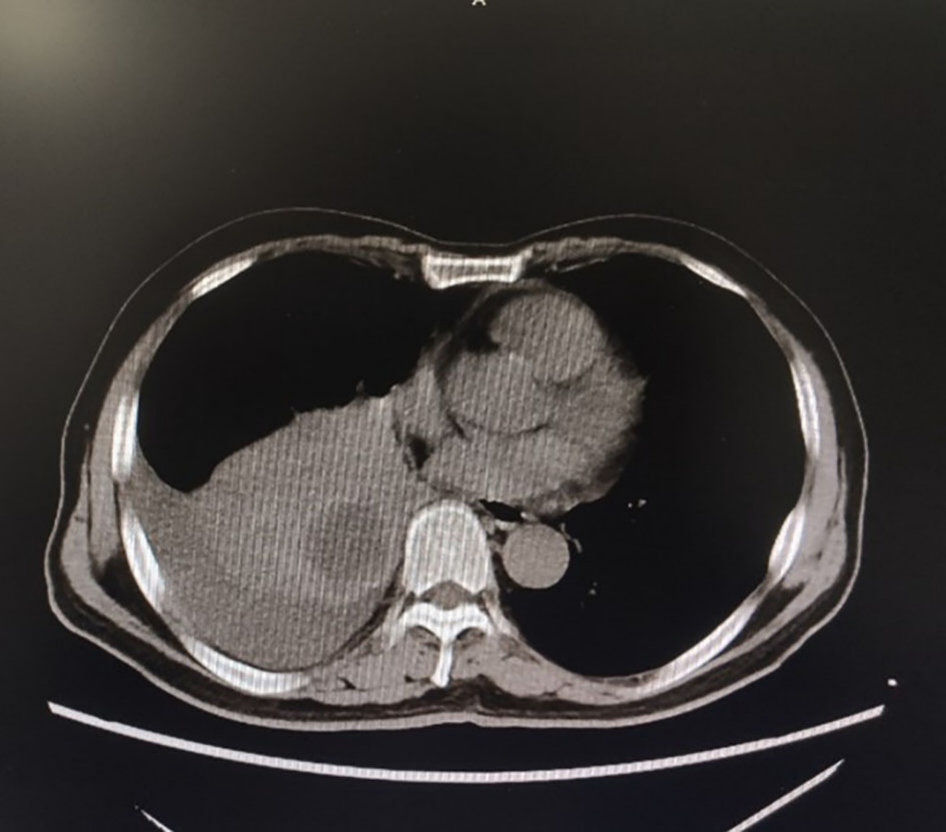
Chest CT for the first time in our hospital showed a space-occupying mass in the lower lobe of the right lung.

**Figure 2:**
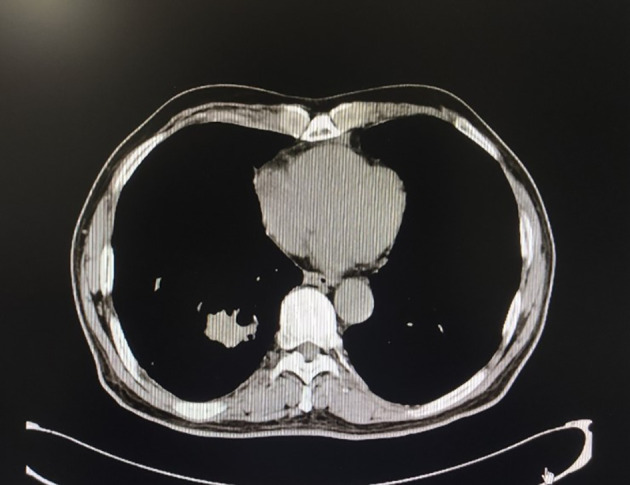
Chest CT after nine cycles of treatment, the space-occupying mass in the lower lobe of the right lung was significantly reduced.

**Figure 3:**
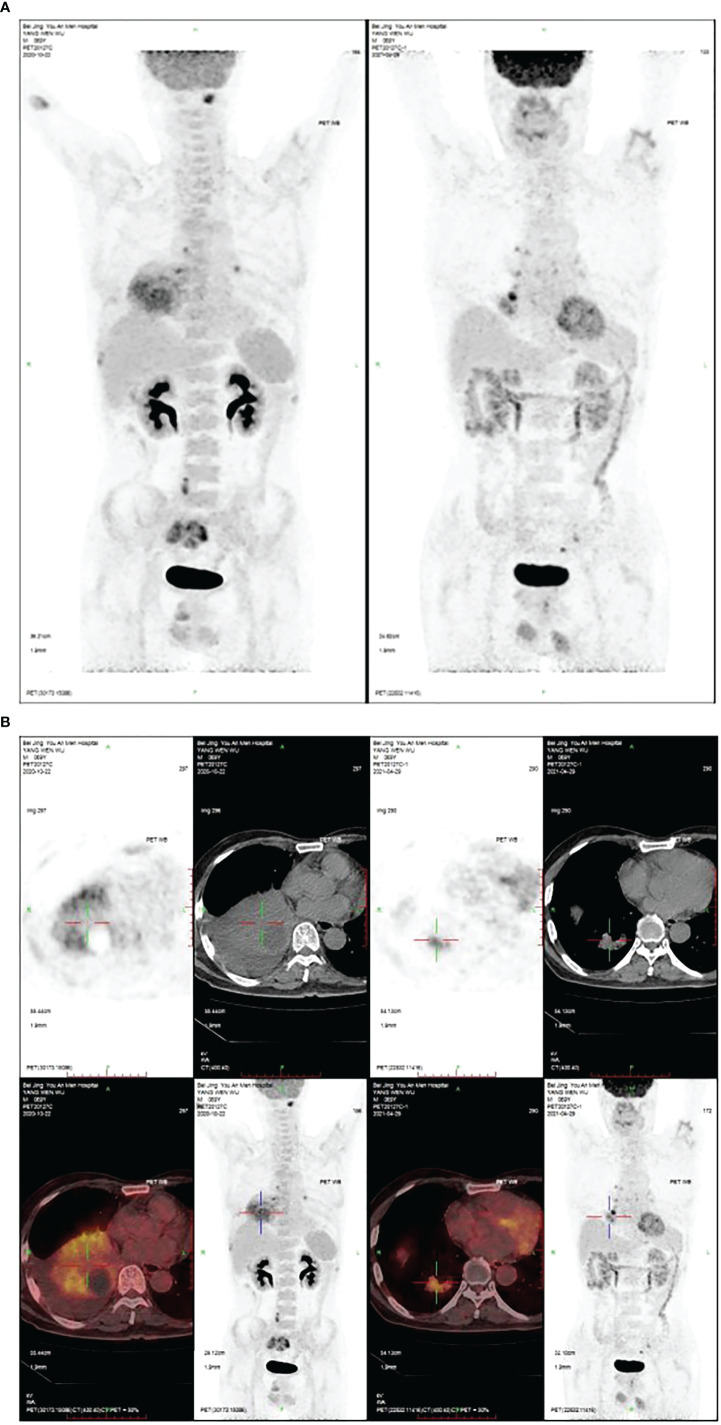
**(A)** After nine cycles of treatment, the PET/CT of the hand and chest showed the space-occupying mass in the lower lobe of the right lung was significantly reduced. **(B)** The PET/CT of hand and chest after nine cycles of treatment. The space-occupying mass in the lower lobe of the right lung was significantly reduced.

**Figure 4:**
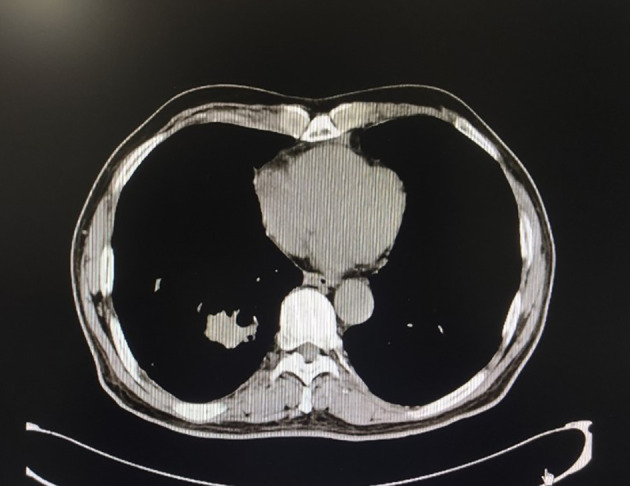
Chest CT one month after surgery.

**Figure 5:**
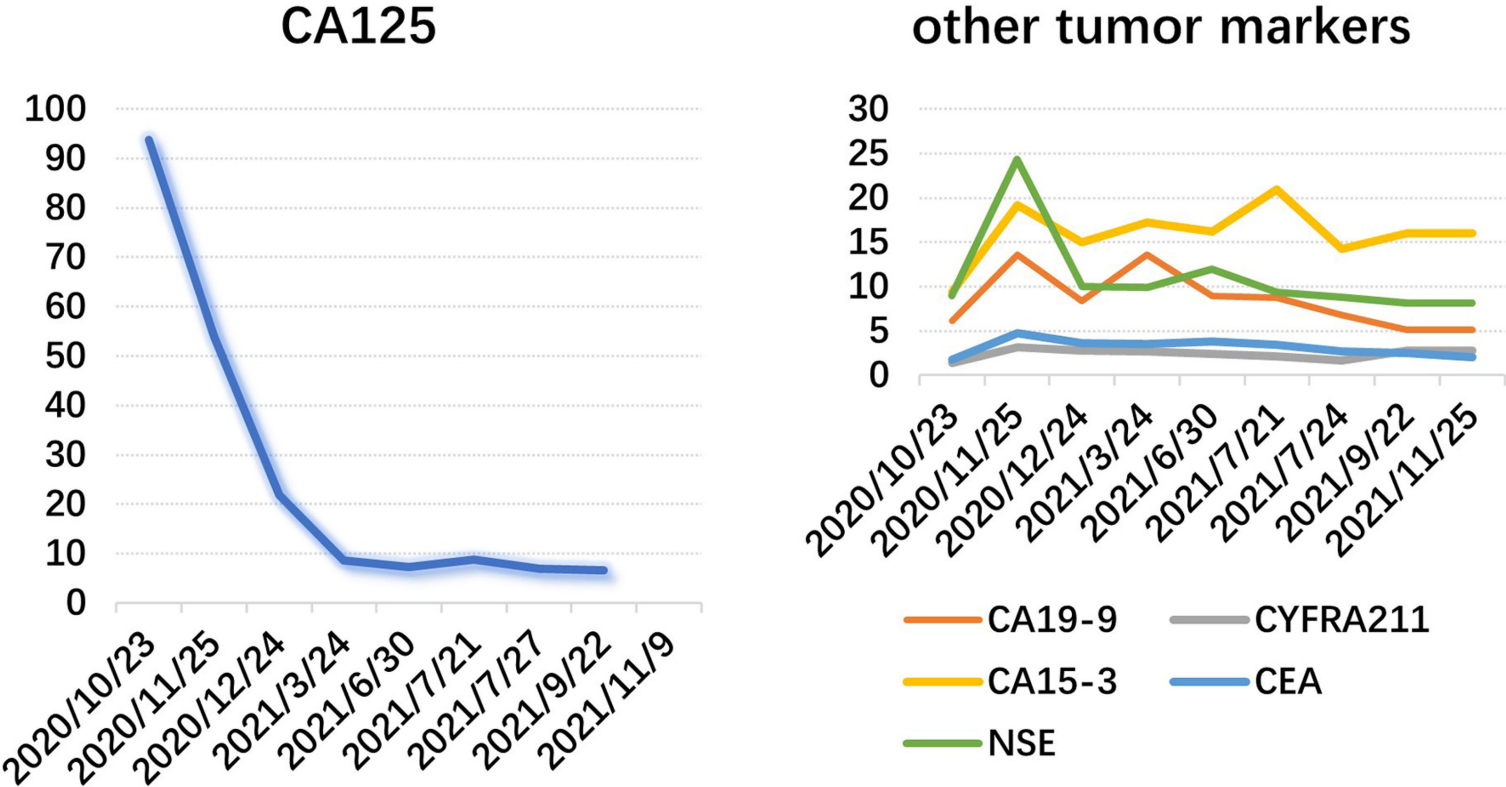
Major abnormal tumor markers.

**Figure 6:**
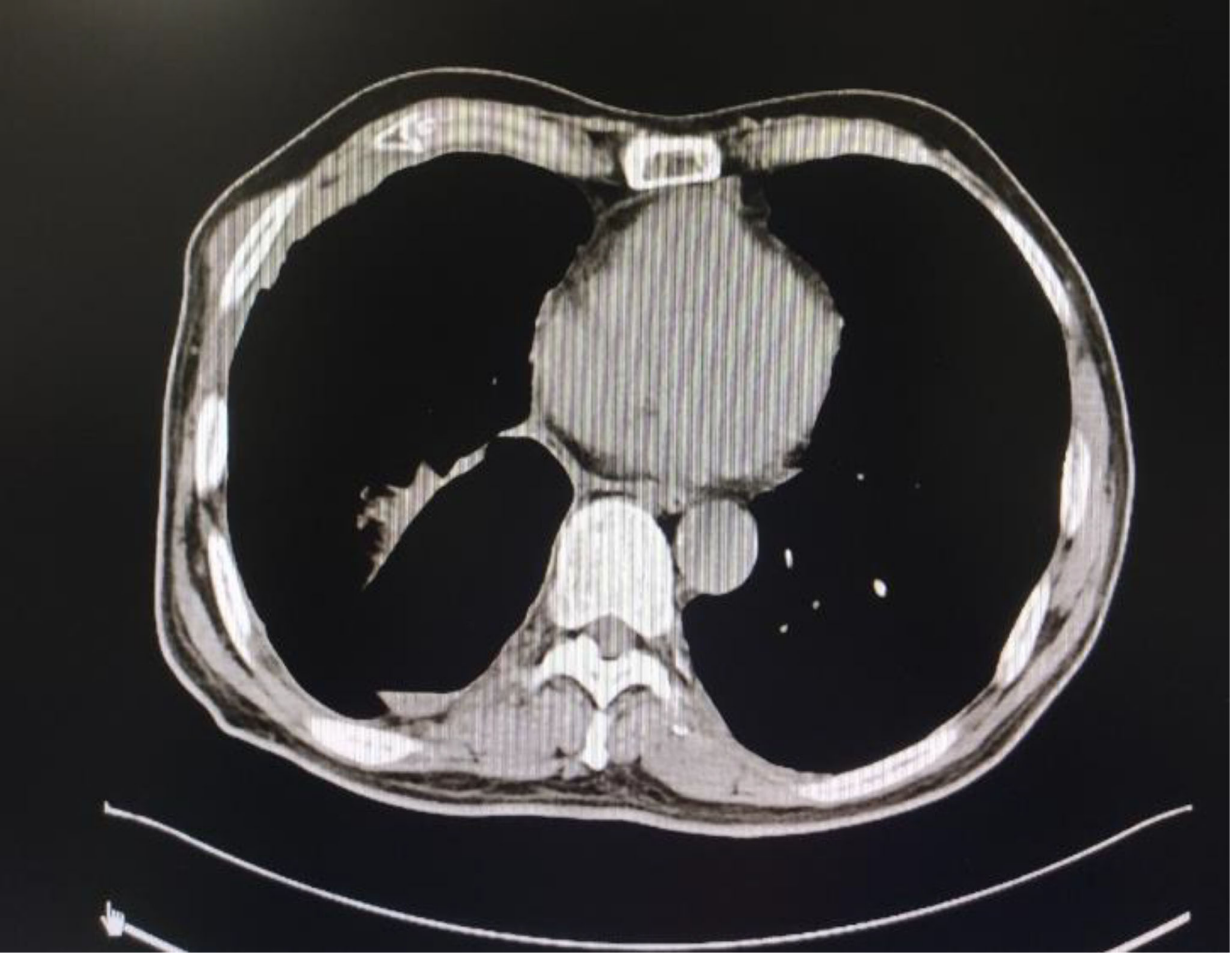
Chest CT after lung surgery and after two cycles of postoperative treatment. The right pleural effusion decreased significantly.

The patient, a 69-year-old male, coughed intermittently with white mucous sputum after catching a cold for more than five months, developed left chest pain for more than two months, and felt wheezing and these symptoms aggravated after activity. Chest CT in the out-patient clinic suggested a tumor in the right lower hilum and multiple military nodules in both lungs and pleura. These findings indicated that central lung cancer should be considered. Pathological consultation revealed adenocarcinoma of the lung. Genetic examination showed EGFR exon20 insertion mutation. Carboplatin plus pemetrexed treatment was not effective, so he was transferred to our hospital. There is nothing special about past history, personal history, and family history. The patient was hoarse, suffocated, and in a wheelchair. PS scores: 2 points. On admission, tumor markers: CA125:93.8U/ml (normal value: 0-24U/ml) and CEA:1.74ng/ml (normal value: 0-5U/ml). Chest CT suggested that the space-occupying lesion in the lower lobe of the right lung was consistent with the manifestation of lung cancer (**Figure 1**). PET/CT: soft tissue mass was seen near the right hilum, FDG uptake increased unevenly, SUVmax9.2; left atlas, right 9th rib, left 10th rib, right humerus and sacrum showed multiple abnormal increased FDG uptake, SUVmax11.6. It was suggested that the hypermetabolic mass adjacent to the right hilum should be considered as the residual metabolic activity of the tumor after the treatment. Brain MRI: no abnormality. Pathological stage: T4N3M1c, stage IVB. Immunohistochemistry of drug sensitivity showed: BRCA-1 (-), ERCC-1 (+), TS (-), MSH-2 (+++), MSH-6 (+++),VEGF(-),PD-L1 (TPS=0). Combined with the examination results, we decided to carry out the first and second cycles of chemotherapy combined with targeted immunotherapy. The specific regimens were as follows: bevacizumab 500mg + pemetrexed 900mg + carboplatin 500mg + durvalumab 1000mg.However, the effect was not good, and the myelosuppression was obvious, so the adjusted regimen was bevacizumab 600mg + pemetrexed 900mg + nedaplatin 100mg + durvalumab 1000mg. After the third cycle of chemotherapy combined with targeted immunotherapy, the patients had few adverse reactions, so we continued to carry on the 4th-9th cycles. After nine cycles of chemotherapy, the re-examination of chest CT (**Figure 2**) and PET/CT showed that the metabolic activity of most of the films in the lower lobe of the right lung increased, SUVmax6.6, significantly reduced from the previous range, and the activity decreased (**Figure 3**). However, there was no change compared with the recent, and the tumor markers did not decrease, so we judged that drug resistance occurred. The relevant examination indexes were in accordance with the surgical indications, so a right lower lobe lobectomy was performed. Examination of postoperative freezing specimens showed: invasive lung adenocarcinoma in the lower lobe of the right lung, with two foci, one moderately differentiated (acinar type 70%, papillary type 20%, solid type 10%), and the other poorly differentiated (solid type 90%, acinar type 10%). The tumor surrounded the bronchial wall and there was a tumor thrombus inside blood vessels. Immunohistochemical results showed: CK7 (+), CK20 (+), TTF-1 (+), NapsinA (weak +), CK5 (-), P40 (-), ALK (D5F3) (-), Ki-67 (hot spot index 15%), P53 (wild type), MLH1 (+), MSH2 (+), MSH6 (+), PMS2 (+), PD-1 (UMAB199) (TILS:20%), NTRK (-). The cutting edge was negative, that is, R0 resection. The patients recovered well after the surgery (**Figure 4**) and received two cycles of postoperative adjuvant chemotherapy combined with targeted immunotherapy. The specific regimens were as follows: bevacizumab 600mg + pemetrexed 900mg + nedaplatin 100mg + durvalumab 1000mg. The tumor marker CA125:6.6 U/ml, CEA:2.0ng/ml, was significantly lower than that before treatment (**Figure 5**). Chest CT (**Figure 6**) suggested that the right pleural effusion decreased significantly after surgery. After two weeks of postoperative treatment, patients with mass elimination, negative lymph nodes, negative bone metastasis, normal tumor markers, PS score: 0, and no indication of progression, had reached CR, so we suspended chemotherapy, used immune maintenance therapy alone, requiring the patient to return regularly. It took one year from the beginning of the treatment to the time that the patient reached CR.

## Discussion and Literature Review

Non-small cell lung cancer accounts for about 85% of lung cancer (29). With the understanding of gene mutation in NSCLC, the emergence of new drugs (30), and the use of immune checkpoint inhibitors, the treatment of NSCLC has improved. For most patients, chemotherapy is still an important part of systemic treatment, but for about 50% of advanced NSCLC patients (31), molecular targeted therapy or immunotherapy instead of chemotherapy is the standard first-line treatment.

According to the immunohistochemical results of the patients, it is suggested that carboplatin, nedaplatin, and pemetrexed can be used and immunotherapy is effective. Therefore, the external hospital regime: carboplatin combined with pemetrexed is selected first, but because of obvious myelosuppression, nedaplatin is used instead, and the effect is remarkable. Although immunohistochemistry indicates the effectiveness of the drug, it is still necessary to flexibly adjust the treatment regimen according to the patient’s condition, to maximize the effect of chemotherapeutic drugs and minimize the adverse reactions, which reflects the necessity of individualized chemotherapy. The nine cycles of chemotherapy before surgery can be carried out smoothly because we provide patients with adequate support treatment and the patient’s systemic condition and immunity are maintained at a good level. Because drug resistance develops after nine cycles of chemotherapy, and the tumor mass becomes small which is in line with surgical indication, surgery is considered. After the operation, the patient has no progress in metastatic focus. In our view, surgery not only removes the primary lesion, but also reduces the possibility of metastasis and recurrence. Postoperative pathology shows tumor heterogeneity and positive vascular thrombus, which provides a reference for further consolidation of chemotherapy.

Cancer patients with specific gene mutations can benefit from targeted therapy. 69% of patients with advanced NSCLC may have potential operable molecular targets (32). Targeted therapy is effective for adenocarcinoma, most of these patients are young and have never smoked (33).. Platinum dual therapy with or without bevacizumab is the most common choice for advanced NSCLC patients who cannot be treated with targeted therapy, which is also the standard first-line treatment. The understanding of tumor immune patterns, including immune escape, makes a breakthrough in the treatment and lays a foundation for the development of treatment in the future. In this case, although VEGF (-), but for non-squamous non-small cell lung cancer, the vascular targeting drug bevacizumab can be used. Attention needs to by being paid to side effects such as hypertension, hemoptysis, albuminuria, and others. As early as 2015, the BEYOND study (34) for the Chinese population confirms that bevacizumab combined chemotherapy can notably prolong PFS, and median total survival time (mOS) compared with chemotherapy alone. Meanwhile, the bevacizumab combined treatment group significantly improved the objective remission rate (ORR) and disease control rate (DCR). In 2018, the State Drug Administration (NMPA) approves the first-line treatment of platinum-containing dual-drug chemotherapy combined with bevacizumab for advanced NSCLC. According to the 2021 Chinese Society of Clinical Oncology (CSCO) guidelines for the diagnosis and treatment of non-small cell lung cancer (35), platinum-containing dual-drug chemotherapy or platinum-containing dual-drug chemotherapy plus bevacizumab (lung squamous cell carcinoma) should be included as first-line treatment for patients with EGFR mutant NSCLC in stage IV, and bevacizumab should be given the first choice for patients with stage IV NSCLC without driving gene and NSCLC. Although this patient has an EGFR mutation, it is specific to exon 20 insertion mutation (EGFR20ins). According to NCCN’s latest guidelines on NSCLC (36): for EGFR20ins NSCLC patients, first-line treatment is chemotherapy combined with immunotherapy; when the disease progresses, targeted drugs, Amivantamab or Mobocertinib, are recommended. Because the effect of neoadjuvant therapy is better, we do not recommend patients take targeted drugs.

Immune checkpoint inhibitors (ICIs) are relatively new immunotherapy-based drugs. Different from traditional chemotherapy drugs, ICIs play a role in enhancing the natural tumor-killing response of the human body. Nivolumab and Pembrolizumab (PD-1 inhibitors), Atezolizumab and Durvalumab (PD-L1 inhibitors) have been approved by FDA in subsequent line therapy for advanced NSCLC patients without the sensitive mutation. They have been shown to improve the survival rate of advanced NSCLC. Although ICIs are not used in the guidelines for first-line treatment of advanced lung cancer patients with low expression of PD-L1, several new reports suggest that chemotherapy combined with immunotherapy as first-line treatment can effectively improve survival, regardless of PD-L1 expression level such as IMpower131、IMpower150. In one report (37), in the PD-L1 < 1% subgroup, the immune plus chemotherapy (I+C) regimen was more effective than chemotherapy in both OS and PFS. In the subgroup with PD-L1 ≥ 50%, the OS and PFS of the I+C regimen were also longer than those of chemotherapy. Therefore, for advanced NSCLC with different PD-L1 expressions, it is recommended to choose PD-1/PD-L1 inhibitors combined with chemotherapy in the first line and pay close attention to adverse events. Therefore, this patient chose chemotherapy combined with PD-L1 as the first choice. During the period of treatment, the tumor decreased significantly, and there were no immune-mediated adverse events in this patient. However, many factors must be considered in the treatment plan, such as therapeutic toxicity.

There is growing evidence that surgical resection is beneficial to survival in selective advanced patients. The surgery prolongs the survival time of some selected patients with stage IV NSCLC (38–40).. A national analysis (41) of long-term prognosis after surgery showed that the 5-year OS of patients with cT1-2, N0-1, M1 or cT3, N0, M1 was superior to non-operative treatment. These data supported surgical resection of specific advanced NSCLC patients. In a study (42) of a case with comprehensive treatment, the surgical prognoses of patients with stage IV NSCLC were analyzed, and the 1 -, 2-and 3-year OS rates were 75.9%, 59.1%, and 42.2% respectively. It is concluded that lung surgery may be a good choice for patients with IV stage NSCLC during comprehensive treatment. Surgical resection of malignant lesions can reduce the tumor load and restore the immune function of patients (43). Even in the case of pleural effusion (pleural dissemination or effusion is a contraindication for surgical treatment of NSCLC), the 5-years of OS after the surgery can reach 33.1%, and the patient recovers well after the surgery, and the primary tumor can be controlled (44). Many data suggest that radical surgery may be an option for symptom relief and further systematic treatment.

Lung cancer has strong temporal and spatial heterogeneity, which will affect its diagnosis and treatment. Understanding the heterogeneity of tumors may lead to new treatments, thus prolonging the survival time of patients with lung cancer in the future. It is the existence of tumor heterogeneity that makes individual differences in treatment methods. After a comprehensive evaluation of this patient, we worked out an accurate and individualized treatment plan; different treatment methods were given to different reactions due to individual differences in the process of treatment. The difference in pathological type and location of the patients determined the type of operation, which reflected the importance of individualized treatment. In short, the future treatment of lung cancer is inseparable from individualization.

## Conclusion

The incidence and mortality of lung cancer are still high. Therefore, improving the therapeutic effect of lung cancer patients is an urgent task. In-depth study and understanding of tumor heterogeneity, continuing to find new treatment methods and individualized treatment for patients, will further improve the treatment outcome of lung cancer.

## Author Contributions

All authors contributed to the article and approved the submitted version.

## Conflict of Interest

The authors declare that the research was conducted in the absence of any commercial or financial relationships that could be construed as a potential conflict of interest.

## Publisher’s Note

All claims expressed in this article are solely those of the authors and do not necessarily represent those of their affiliated organizations, or those of the publisher, the editors and the reviewers. Any product that may be evaluated in this article, or claim that may be made by its manufacturer, is not guaranteed or endorsed by the publisher.
